# BrainAgeMS: protocol for a prospective comparative study of brain aging in healthy adults and people with multiple sclerosis

**DOI:** 10.3389/fneur.2026.1899641

**Published:** 2026-08-11

**Authors:** Sebastián Navarrete, Milena Capiglioni, Stefanie Marti, Lukas Pirpamer, Richard McKinley, Robert Hoepner, Alejandro León Betancourt, Piotr Radojewski

**Affiliations:** 1 Faculty of Engineering, Universidad de Concepción, Concepción, Chile; 2 Support Center for Advanced Neuroimaging, Institute for Diagnostic and Interventional Neuroradiology, University of Bern, Bern, Switzerland; 3 Translational Imaging Center (TIC), SITEM-INSEL, Bern, Switzerland; 4 High-Field MR Center, Max Planck Institute for Biological Cybernetics, Tübingen, Germany; 5 Department of Neurology, Inselspital, Bern University Hospital and University of Bern, Bern, Switzerland

**Keywords:** 7 T MRI, brain aging, chemical exchange saturation transfer, multiple sclerosis, neurodegeneration, quantitative MRI, T1 relaxometry

## Abstract

Biological brain aging can diverge substantially from chronological aging, particularly in chronic neurological conditions like multiple sclerosis (MS), where neuroinflammatory and neurodegenerative processes may accelerate age-related brain changes. This study aims to determine whether multipool Chemical Exchange Saturation Transfer (CEST) and T1 relaxometry at 7 T can serve as biologically grounded imaging markers of brain age in healthy adults and people with MS (pwMS). BrainAgeMS (NCT06221631) is a prospective, single-center, cross-sectional comparative study recruiting 200 participants aged 18–65 years: 100 healthy controls (HC) and 100 pwMS. All participants undergo blood sampling for biological age determination using the PhenoAge algorithm, motor, cognitive, and psychological assessments, and 7 T MRI featuring multipool CEST and T1 relaxometry. A brain age prediction model trained on HC data will be applied to pwMS to derive a brain age gap. The primary outcomes are the two brain-age gaps in pwMS: the differences between estimated brain age and (i) chronological age and (ii) PhenoAge. Secondary outcomes include associations of these gaps with disability, cognition, fatigue, and quality of life. If validated, these quantitative MRI markers could complement existing tools for MS disease monitoring and serve as outcome measures for future brain health interventions.

**Clinical Trial Registration:**

https://clinicaltrials.gov/study/NCT06221631, identifier NCT06221631

## Introduction

Aging is a natural, complex, and multifactorial process that results in changes in the body’s structure, function, and chemistry over time, at both the organ and whole-organism levels. As society’s age distribution shifts dramatically, population aging is increasingly recognized as a crucial societal challenge (1), with the burden appearing even higher among individuals with chronic diseases, particularly neurological disorders (2). Significant differences in life expectancy worldwide, and even within local communities, indicate that aging is not a purely temporal process. Biological or organ-specific aging can differ substantially from chronological aging (measured by date of birth) (3). This distinction underscores the need for methods to quantify the age of specific target organs, which is essential for developing preventive measures and effective treatment strategies for age-related conditions.

Currently, there is no standard method for assessing biological age. Various approaches have been explored, including leukocyte telomere length (LTL) and biomarker-based algorithms that incorporate blood chemistry and clinical data (4). However, predicting organ-specific biological age remains an unmet need. Objective measurement of brain age and its relationship to chronological age could improve risk prediction for age-related cognitive and motor decline.

Advanced imaging techniques and diverse neuroimaging modalities offer promising avenues for more accurate estimation of brain age. In particular, ultra-high-field MRI (7 T) with T1 relaxometry and chemical exchange saturation transfer (CEST) may provide deeper insight into brain aging (5). Unlike standard MR images, the image intensities in quantitative T1 relaxometry maps represent absolute measures of tissue properties with physical units, with significant determinants including changes in myelin, iron, and water content, as well as inflammatory activity.

Chemical exchange saturation transfer imaging adds a molecular layer to ultra-high-field MRI by leveraging exchange and cross relaxation between bulk water and labile protons in endogenous solutes, producing a measurable reduction of the water signal that can be decomposed into distinct pools via multipool modeling (6, 7). In the context of aging, this is attractive because it targets mechanisms that plausibly drive brain biological age, including loss of proteostasis and shifts in the macromolecular and lipid environment (8). Our acquisition and analysis focus on the amide, the amine, the aliphatic relayed NOE, and the semisolid magnetization transfer pools, which together provide complementary sensitivity to mobile proteins and peptides, mixed metabolite and macromolecular amine chemistry, membrane and myelin-associated lipids, and overall macromolecular density (9).

A core target is the amide pool, which reflects mobile proteins and peptides and is influenced by microenvironmental factors such as pH, linking it to age-related loss of proteostasis and altered tissue homeostasis (10, 11). In line with this biological rationale, multipool CEST at 7 T demonstrated that the amide pool amplitude in gray matter decreases significantly with age and presents the strongest age-related effect among the evaluated CEST pools at 7 T (5). More recently, using the same multipool acquisition and analysis protocol applied in the present study, Capiglioni et al. (12) confirmed robust negative correlations between amide signal and age in both cortical and deep gray matter at 7 T, whereas corresponding age effects at 3 T were substantially weaker, highlighting the added value of ultra-high-field CEST for detecting age-related molecular changes. The amine pool provides complementary sensitivity to exchangeable amine protons from small metabolites and amino acid chemistry, which are expected to vary with age through shifts in neurotransmitter cycling and metabolic balance (13, 14). Upfield rNOE contrast is linked to mobile macromolecules, including lipid and protein environments, with strong WM sensitivity, making it a plausible readout of age-related myelin remodeling (9, 15). Importantly, rNOE amplitudes have also been shown to decrease significantly with age in gray matter at 7 T and exhibited robust negative age correlations in both gray matter and deep gray matter in subsequent validation studies, further supporting their relevance as markers of molecular brain aging (5, 12). The MT pool captures the semisolid macromolecular background that is tightly connected to myelin and tissue microstructure, and MT measures show small but significant age-related reductions in normal-appearing white matter (NAWM).

While chronological age, biological age, and brain age are assumed to correlate in highly selected healthy individuals, these parameters may diverge considerably in the general population due to multiple factors (16). Among them are chronic neurological conditions of the central nervous system (CNS), as these seem to exacerbate the burden of aging processes in the brain (2). Consistently, brain-age predictor models have been widely applied across several chronic neurological and psychiatric conditions to evaluate structural age acceleration, including Alzheimer’s disease (17, 18), mild cognitive impairment (17, 19), and Parkinson’s disease (18). Multiple sclerosis (MS) is another example of such a condition, where neuroinflammatory and neurodegenerative processes exacerbate age-related structural changes (20). It thus represents a compelling model to investigate the interaction between aging and chronic neurological disorders. Following this rationale, the BrainAgeMS study (NCT06221631) aims to estimate whether multipool CEST, which has been shown to be sensitive to age, and T1 relaxometry (i) can predict brain age, which is defined to be equal to the chronological age in healthy adults, and whether (ii) pwMS exhibit a positive age gap relative to both chronological age and PhenoAge.

## Methods and analysis

### Study design

BrainAgeMS (NCT06221631) is a single-center, cross-sectional, case–control study designed to evaluate quantitative MRI markers for brain age prediction and to investigate the effect of MS on brain aging. Two hundred participants aged 18–65 are recruited, comprising 100 healthy controls and 100 pwMS. Participants do not receive financial compensation for study participation. The study is conducted in the Neurology Department at Inselspital, Bern, and at SITEM, Bern. Both sites are part of University Hospital Bern, with clinical assessments performed at the Department of Neurology and 7 T MRI examinations performed at SITEM.

### Participants

The study aims to recruit 100 healthy controls (HC) and 100 pwMS, aged 18–65 years. To ensure a uniform age distribution, recruitment targets for both cohorts are stratified into four age categories: 18–30 years (25–27 participants), 31–40 years (21–23 participants), 41–50 years (21–23 participants), and 51–65 years (29–31 participants). These age categories are used solely for recruitment stratification, and age is analyzed continuously. As no prior data are available on 7 T MRI-based brain age assessment in this population, a formal sample size calculation is not feasible. The proposed sample size is based on feasibility considerations and the objective of establishing a comprehensive normative brain age reference dataset across the adult lifespan.

### Inclusion and exclusion criteria

To establish a normative brain age dataset suitable for the study of CNS diseases such as MS, healthy control recruitment follows stringent selection criteria to ensure consistency across age-related parameters. Only participants who demonstrate close alignment between biological and chronological age undergo MRI. This approach aims to minimize age-related variability in the HC cohort and to establish robust reference parameters for age estimation using 7 T MRI.

For feasibility, biological age is calculated using the PhenoAge model (21). The PhenoAge model is trained on NHANES III data (United States, 1988–1994) and calculates the phenotypic age, defined as the expected age within the population that corresponds to a person’s estimated mortality risk (21). For HC recruitment, only participants with a deviation of −8 to +1 years between the calculated PhenoAge and the chronological age proceed to the MRI examination phase. This range is defined pragmatically, based on preliminary unpublished data from a subgroup of participants rather than a previously validated external standard, and allows a correction both for differences between the United States and Switzerland and for the substantial increase of life expectancy worldwide since the time period used for the training of the model (22). For study and budget feasibility, we conservatively estimate a potential exclusion rate of up to 50% under this criterion, ensuring the protocol remains viable even under strict screening conditions.

For pwMS recruitment, eligible participants have a confirmed diagnosis of MS of any subtype and an EDSS score of 6.0 or lower. Participants who experienced a clinical relapse within 6 months prior to screening are excluded, ensuring that the pwMS cohort comprises clinically stable patients without recent relapse. Medications and any comorbidities not addressed by the exclusion criteria are systematically collected and documented in the case report form (CRF) for all participants. Inclusion and exclusion criteria are presented in Tables 1 and 2, respectively.

**Table 1:** Inclusion criteria.

Criteria	HC cohort	pwMS cohort
Age	18–65 years	18–65 years
Language	Sufficient German or French skills	Sufficient German or French skills
Informed consent	Written informed consent	Written informed consent
Disease status	No known neurological disease (except primary headaches)	Confirmed diagnosis of multiple sclerosis (any subtype)
Disability level	Not applicable	EDSS ≤6.0

**Table 2:** Exclusion criteria.

Criteria	HC cohort	pwMS cohort
Neurological disease	Any neurological disease (except primary headaches)	Any neurological disease other than MS (except primary headaches)
Recent relapse	Not applicable	Clinical relapse within 6 months prior to screening
BioAge screening	PhenoAge–chronological age difference outside the range −8 to +1 years	Not applicable
Chronic disease	Any other chronic progressive disease	
Body mass index	BMI > 30 kg/m^2^	
Pregnancy/Lactation	Excluded	
Smoking	History within 10 years prior to recruitment	
Substance use	Any recreational drug use (except moderate alcohol intake <10 g/day or prescribed medical cannabis)	
Head trauma	Previous head trauma with known/suspected intracranial consequences	
MRI contraindications	Active implants	
	Passive ferromagnetic implants	
	Passive non-ferromagnetic metallic implants >4 cm in RF coil region	
	Large tattoos in RF coil region	
	Claustrophobia	
	Suspected non-compliance	

### Withdrawal and discontinuation criteria

Participants may withdraw from the study at any time, and any data collected up to that point may still be used in coded form. Discontinuation criteria include:

- Withdrawal of informed consent.
- A change in health status that renders continued participation unwise, or that newly meets an exclusion criterion.
- Safety concerns for any reason.
- Evidence or suspicion of harm from any planned study intervention or action.
- Changes in accepted 7 T MRI safety guidelines.
- Inability to remain sufficiently still for the required scan duration.

Withdrawn or discontinued participants are replaced by additional volunteers to maintain the target sample size.

### Study schedule

The study timeline consists of a recruitment visit (up to 21 days prior) and a primary study visit (Day 0). The primary study visit encompasses all clinical procedures, including vital parameters, physical examinations, functional and neuropsychological assessments, and participant-reported outcome measures (PROMs). For both cohorts, 7 T MRI data acquisition is planned to take place on the same day as the primary study visit. If this is not feasible, a separate MRI visit is scheduled within 21 days of the primary study visit. For HC specifically, an additional blood sample is collected at the recruitment visit to confirm PhenoAge-based eligibility before the study visit is scheduled. The content of visits varies depending on the cohort, as summarized in detail in Table 3.

**Table 3:** Study visit schedule and assessment.

Assessment	Recruitment	Study visit	MRI visit
Time window	Up to 21 days prior	Day 0	Within 21 days post
Participant information	X, Y		
Inclusion/Exclusion criteria	X, Y		
Informed consent	X, Y		
Medical screening and blood sampling			
Vital parameters		X, Y	
Adverse event monitoring		X, Y	X, Y*
Blood sampling	Y	X, Y	
Clinical assessment			
Expanded Disability Status Scale		X	
Floor Sitting-to-Rise Test		X, Y	
25-Foot Walk Test		X, Y	
Nine-Hole Peg Test		X, Y	
Neuropsychological assessment			
Brief international cognitive assessment for multiple sclerosis		X, Y	
Participant-reported outcome measures			
Beck Depression Inventory-II		X, Y	
Fatigue Severity Scale		X, Y	
Epworth Sleepiness Scale		X, Y	
WHO Quality of Life-BREF		X, Y	
Multiple Sclerosis Impact Scale-29		X	
7 T MRI		X, Y	X, Y*

X: pwMS cohort. Y: HC cohort. *If 7 T MRI cannot be performed during the main study visit.

### Data acquisition

#### Blood samples

All participants undergo blood sampling for calculation of the biological age (PhenoAge model (21)) and for biobanking for future projects. This study uses the clinical-biomarker-based phe notypic age estimate. No DNA methylation measurement or meth ylation-based processing is performed. The following blood parameters are assessed for PhenoAge: albumin, fasting glucose, creatinine, alkaline phosphatase, c-reactive protein, white blood cell count, blood lymphocyte (count and percentage), mean volume of red blood cells, red blood cell width glycated hemo globin (HbA1c), total cholesterol, blood urea nitrogen, and cyto megalovirus IgG titers (21).

#### Clinical assessments

##### Expanded Disability Status Scale

The Expanded Disability Status Scale (EDSS) is calculated based on the neurological examination of pwMS and quantifies disability in MS, with a score ranging from 0 (no disability) to 10 (death from MS). EDSS steps 1.0 to 4.5 refer to patients who are fully ambulatory with no or mild functional impairment, whereas steps higher than 4.5 are characterized by increasing impairment in walking capacity (23).

##### Floor Sitting-to-Rise Test

The Floor Sitting-to-Rise Test (fSRT) evaluates the ability to transition between standing and sitting on the floor. Scored on a 10-point scale, the test integrates measures of strength, flexibility, balance, and coordination and has been shown to be a significant predictor of functional independence and all-cause mortality in middle-aged and older adults (24, 25).

##### 25-Foot Walk Test

25-Foot Walk Test (25-FWT) is a standardized measure of short-distance walking speed in which participants are instructed to walk 25 feet as quickly and safely as possible. As a core component of the Multiple Sclerosis Functional Composite (MSFC) (26), it is widely used to quantify ambulatory function in individuals with multiple sclerosis, with changes of approximately 20% considered clinically meaningful (27).

##### Nine-Hole Peg Test

Nine-Hole Peg Test (9-HPT) is a standardized measure of upper extremity function in which participants are required to place and remove nine pegs from a pegboard as quickly as possible and is also part of the MSFC. It is widely used to assess manual dexterity in individuals with multiple sclerosis, with changes of approximately 20% considered clinically meaningful (27).

### Neuropsychological assessments

#### Brief International Cognitive Assessment for Multiple Sclerosis

The Brief International Cognitive Assessment for Multiple Sclerosis (BICAMS) is a standardized cognitive screening battery comprising the Symbol Digit Modalities Test (SDMT), the Brief Visuospatial Memory Test–Revised (BVMT-R), and a validated verbal learning and memory test, designed to assess cognitive domains frequently affected in MS such as information processing speed, mental flexibility, visuospatial memory, and verbal episodic memory and learning (28).

SDMT measures information processing speed and working memory (29), providing excellent psychometric properties (30). The test consists of a key on top of a sheet in which abstract symbols are paired with single digits from zero to nine. Below this key, the abstract symbols are displayed pseudo-randomly in several rows without digits. The participant is asked to orally report the corresponding digits of each symbol as quickly as possible. The number of correct responses within 90 s is measured.

BVMT-R (31) was developed for assessing visuospatial memory. The participant is confronted during each training trial with the same stimulus template of six geometric figures for 10 s. After this period, the template is removed, and the participant is asked to draw all figures with correct shape and correct position from memory. For BICAMS, only the three learning trials are mandatory, while the delayed recall and recognition trials are optional, though this study systematically collects the delayed recall trial for all participants.

For assessment of verbal short-term memory and learning, the California Verbal Learning Test–II (CVLT-II) was originally included (32, 33). The International Multiple Sclerosis Cognition Society (IMSCOGS) committee agreed that in non-English-speaking countries, varying instruments for verbal learning and memory may be performed if those have been validated (28). For this study, we used the German BICAMS version, which uses the validated German version of the Rey Auditory Verbal Learning Test (verbaler Lern- und Merkfähigkeitstest, VLMT (34)). As most participants for this trial are German-speaking, and it has been shown that the combination of SDMT and BVMT-R is the most important for the detection of MS-related cognitive disorders (35), we only perform the complete BICAMS in its German version, while French-speaking individuals do not perform any verbal learning and memory tests.

#### Participant-reported outcome measures

##### Beck Depression Inventory

The Beck Depression Inventory-II (BDI-II) is a 21-item self-report questionnaire designed to assess depressive symptoms, covering both cognitive and physical aspects (36). Each item is rated on a 4-point scale (0–3 points, score range 0–63 points). A score of 14 or higher indicates the presence of depressive symptoms, categorized as mild (14–19 points), moderate (20–28 points), or severe (≥29 points), while scores of 0–13 are considered minimal.

##### Fatigue Severity Scale

The Fatigue Severity Scale (FSS) is a 9-item self-report questionnaire designed to assess the impact of fatigue in daily life (37). The FSS evaluates the effects of fatigue on motivation, physical activity, work, family life, and social functioning. Each item is rated on a 7-point scale, and the total score is calculated as the mean of the item scores. Scores of 4 or higher indicate clinically significant fatigue.

##### Epworth Sleepiness Scale

The Epworth Sleepiness Scale (ESS) is a brief, self-administered questionnaire that quantifies the likelihood of falling asleep in everyday situations, differentiating it from general tiredness (38). The ESS comprises eight items, each scored on a 4-point scale, yielding a total score ranging from 0 to 24. Higher scores indicate greater daytime sleepiness, with scores above 10 suggesting excessive sleepiness.

##### WHO Quality of Life–BREF

The World Health Organization Quality of Life–BREF (WHOQOL-BREF) is an abbreviated version of the WHOQOL-100, a widely used instrument for assessing a person’s self-perceived quality of life over the past 2 weeks. This 26-item questionnaire assesses quality of life across four key domains: physical well-being (7 items), psychological health (6 items), social relationships (3 items), and environment (8 items) (39). In addition, it includes two general questions about the overall quality of life and general health. Respondents rate each item using a 5-point scale. The domain scores are then computed and scaled to 0–100, facilitating straightforward interpretation.

##### Multiple Sclerosis Impact Scale-29

Multiple Sclerosis Impact Scale-29 (MSIS-29) is an MS-specific patient-reported outcome measure designed to evaluate how MS affects everyday life from the patient’s perspective (40). The questionnaire comprises two subscales: a physical impact section with 20 items addressing functional and physical limitations, and a psychological impact section with nine items capturing the emotional and mental burden of the disease. Each item is rated on a 5-point Likert scale ranging from 1 (not at all) to 5 (significantly), with subscale scores commonly transformed to a 0–100 scale.

### MRI

#### 7 T MRI acquisition

All MRI examinations are performed at the MRI visit (Table 3) on a 7 T Terra system (Siemens Healthineers, Erlangen, Germany) using a 32-channel receive/1-transmit head coil. All sequences are performed in clinical mode (single transmit).

**Figure 1:**
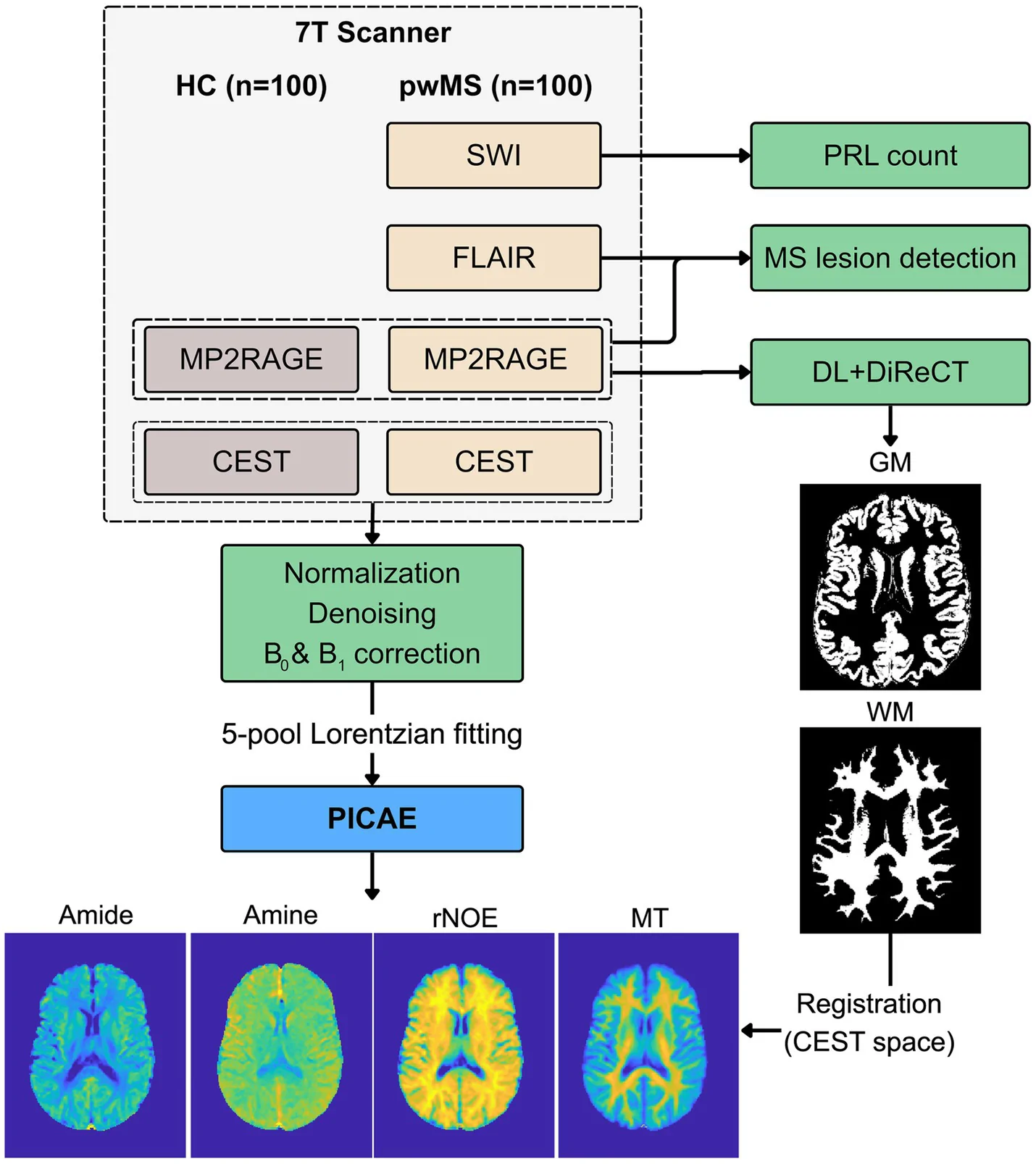
Overview of the 7 T MRI acquisition and processing pipeline. All participants undergo MP2RAGE and CEST imaging. SWI and FLAIR are acquired only for the pwMS cohort. CEST, chemical exchange saturation transfer; DL + DiReCT, a deep-learning neuroanatomy segmentation, followed by diffeomorphic registration-based cortical thickness; FLAIR, fluid-attenuated inversion recovery; GM, gray matter; HC, healthy controls; MP2RAGE, magnetization-prepared 2 rapid gradient echoes; MS, multiple sclerosis; MT, magnetization transfer; PICAE, physics-informed conditional autoencoder; PRLs, paramagnetic rim lesions; pwMS, people with multiple sclerosis; rNOE, relayed nuclear Overhauser effect; SWI, susceptibility-weighted imaging; WM, white matter.

The core imaging protocol consists of Magnetization-Prepared 2 Rapid Acquisition Gradient Echoes (MP2RAGE) for T1 relaxometry and high-resolution anatomical imaging and 3D snapshot GRE CEST (41). Fluid-attenuated inversion recovery (FLAIR) and susceptibility-weighted imaging (SWI) are only acquired for pwMS to detect white matter (WM) lesions as part of routine clinical practice for pwMS (see Figure 1 for an overview of the acquisition and postprocessing pipeline).

CEST imaging is performed according to a previously established protocol for reliable multipool evaluation (5). The presaturation module applies two average B1 pulse amplitudes (0.72 and 1.00 μT) and samples 55 frequency offsets, spaced unevenly from −100 to +100 ppm. A densely sampled frequency range between −5 and +5 ppm is used, with 39 offsets, and two reference scans are acquired at −300 and +300 ppm. Image acquisition has a voxel size of 0.9 × 0.9 × 3 mm and an FOV of 186 × 230 mm^2^. Quantification of CEST contrasts is performed using five-pool Lorentzian fitting (water, amine, rNOE, amide, and magnetization transfer) within a physics-informed conditional autoencoder pipeline (42). Z-spectra are denoised, normalized, and corrected for B0 and B1 inhomogeneities before fitting (5).

#### Assessment of white matter lesions and paramagnetic rim lesions

MS WM hyperintensities are identified using automated or semi-automated MRI analysis pipelines that can leverage multiple image contrasts, such as T2-FLAIR and MP2RAGE, to better distinguish lesions from normal brain tissue. As part of the exploratory methodological analysis of this study, we will evaluate various automated segmentation tools. In a preliminary proof of concept, we previously used FLAMeS (43) and DeepSCAN (44) on T2-FLAIR contrasts for MS lesion segmentation (45). The process includes standard image preparation, computer-assisted lesion detection, post-processing, and final visual inspection by trained experts. In addition, WM changes in healthy control participants are assessed to characterize lesions associated with normal aging, using the same standardized and quality-controlled analysis framework. Further, in pwMS, paramagnetic rim lesions (PRLs) are assessed by an expert based on a visual rating on the SWI sequence.

#### Regional evaluation

Automatic regional brain segmentation is used to derive cerebral WM and gray matter masks using an in-house deep learning model (DL + DiReCT) (46). Quantitative T1 values are extracted in WM, gray matter, and, in pwMS, NAWM. To enable regional quantification of CEST pool metrics (amide, amine, MT, rNOE) in native space, rigid linear registration is applied to align the relevant image contrasts and masks. All segmentations and registrations undergo final visual quality control, with manual correction when necessary.

### BrainAgeMS model

The BrainAgeMS model predicts brain age, defined to be equal to chronological age for HC, based on brain imaging parameters.

*Model selection and training*: Using 75% of the HC group, we will explore different modeling approaches from linear regression to various types of regression neural networks and use unsupervised methods such as Principal Component Analysis (PCA) or clustering for dimensionality reduction and variable selection. To assess the robustness of the model selection procedure, the model’s ability to generalize, and its tendency to overfit, we will use resampling techniques.

*Model testing*: The selected model is then tested with the remaining 25% of the HC cohort. The performance of this model is assessed relative to the performances seen during the training and validation phase, both in terms of prediction error (absolute compared to prediction errors seen in the various models assessed in the training/validation phase) and generalizability (relative to the training/validation error of the selected model).

### Statistical analysis plan

#### Sample size

As this is a proof-of-concept study without previous information regarding brain age on this type of quantitative image, a precise sample size cannot be determined. This study aims at providing estimates for the future sample size calculation in clinical studies using the developed methods.

#### Primary outcome analysis

The primary endpoint is the brain age gap, defined as the difference between the estimated brain age and both biological and chronological age in pwMS. Brain age in pwMS will be estimated by a model that predicts chronological age of HC based on regional CEST pools and T1 relaxation data, following the paradigm that brain age equals chronological age in healthy subjects.

The final model is then used to calculate the brain age of pwMS. Calculated brain age in pwMS is compared to chronological and PhenoAge to identify an age gap. To test for a significant deviation from 0 (0 indicating no brain age gap), comparative statistics regarding the distribution of the brain age gap are used.

#### Secondary outcome analysis

The results from the PROMs (BDI-II, FSS, ESS, WHOQOL-BREF, and MSIS-29), clinical assessment (EDSS, fSRT, 25-FWT, 9-HPT), and the neuropsychological assessment (BICAMS) are used to assess the relationship between them and the brain age gaps using a regression analysis adjusted for factors such as sex and chronological age. To control for multiple comparisons among these secondary outcomes, the Holm-Bonferroni method will be applied to all *p*-values. Depending on the collected data, additional adjustment for comorbidities or medications not addressed by the exclusion criteria may be considered.

## Discussion

The BrainAgeMS study (NCT06221631) presents a prospective, single-center, cross-sectional protocol to investigate brain aging in healthy adults and people with multiple sclerosis using 7 T multipool CEST imaging and quantitative T1 mapping. Most published brain age models to date rely on conventional structural T1-weighted MRI, typically using voxel-wise imaging features or morphometric measures such as cortical thickness and volumetry (19, 47). In multiple sclerosis, these structural MRI–based brain age approaches have shown associations with disability progression and disease burden (20). Although brain morphometry research has advanced the understanding of aging processes, current group-based analyses cannot be easily transferred to individual patient-specific analysis.

A key strength of the present study is the acquisition of a large, well-characterized cohort at ultra-high field strength, including 100 healthy controls and 100 people with multiple sclerosis. In particular, the availability of 100 healthy participants scanned at 7 T provides a valuable normative reference across the adult lifespan, which remains scarce for quantitative MRI applications at ultra-high field.

In this context, our study focuses on biologically interpretable quantitative MRI markers, with a primary emphasis on multipool CEST imaging at 7 T. CEST characterizes molecular exchange processes between bulk water and endogenous protons, providing sensitivity to proteins, metabolite-related chemistry, and macromolecular environments (6, 14). Multipool modeling separates distinct signal contributions, such as amide, amine, relayed NOE and magnetization transfer, enabling a specific assessment of tissue composition. These contrasts are biologically relevant in the context of aging, as they relate to processes such as alterations in proteostasis and macromolecular organization (8). Prior work at 7 T has demonstrated measurable age-related effects in multipool CEST signals, particularly in the amide pool (5). By capturing these microstructural tissue properties, this quantitative approach offers sensitivity to early tissue alterations that may precede the overt macroscopic atrophy typically measured by conventional structural MRI.

The normative dataset generated in this study will provide effect sizes and variance estimates required for future sample size calculations and model validation. A possible extension of this work would be a longitudinal assessment to understand individual subject trajectories. From a clinical perspective, if the brain age gap derived from quantitative MRI proves to be robust and reproducible, it could (A) complement existing markers of neurodegeneration in multiple sclerosis and support earlier identification of patients at risk of accelerated disease progression (20), potentially guiding timely adjustments to disease-modifying therapies, and (B) serve as a potential outcome measure in future studies on brain health in general, such as evaluating the efficacy of treatments or lifestyle interventions in aging populations.

Several limitations must be considered. First, the study uses PhenoAge to screen healthy controls and as a reference for biological age comparison. While PhenoAge is associated with morbidity and mortality risk (21), aging is widely recognized as a multidimensional and tissue-specific process that cannot be fully captured by a single biomarker (48, 49). Consequently, both the HC selection criterion and the comparison between MRI-derived brain age and blood-based biological age should be interpreted with caution. Second, the asymmetric PhenoAge exclusion threshold (−8/+1 years, Table 2) was designed to exclude accelerated biological aging from the HC cohort while retaining participants who are biologically younger than their chronological age (as expected in a healthy cohort). This may narrow the resulting normative model toward an optimally aging reference rather than the full range of biological aging seen in the general population, a trade-off that should be considered when interpreting the resulting normative model. Third, no prior normative data exist for 7 T quantitative MRI–based brain age estimation in this population. As a result, a formal sample size calculation was not feasible, and the cohort size of 200 participants was defined based on feasibility considerations and the objective of establishing a reference dataset across the adult lifespan, consistent with early-stage biomarker development (50).

Despite these limitations, the proposed framework offers significant advantages for studying both natural brain aging and neurological disease. In the context of general brain health, physiological aging involves subtle macromolecular and microstructural shifts that conventional structural MRI often fails to detect early on. Quantitative MRI measures are uniquely sensitive to these diffuse alterations, providing a tool to monitor healthy aging trajectories and track early signs of age-related cognitive decline. Similarly, in multiple sclerosis, tissue alterations in NAWM are known to occur even in clinically stable patients and may precede overt lesion formation and global atrophy (51). PRLs, reflecting chronic active lesions with ongoing inflammation and iron accumulation, further illustrate the importance of capturing pathological processes beyond focal lesion burden (52). In this broader context, a brain age framework incorporating quantitative MRI parameters may provide a complementary, biologically grounded marker of overall brain integrity, sensitive to both natural aging trajectories and occult pathological processes in patients with mild or stable disease.
